# Consistency between Treatment Effects on Clinical and Brain Atrophy Outcomes in Alzheimer’s Disease Trials

**DOI:** 10.14283/jpad.2023.92

**Published:** 2023-07-13

**Authors:** M. ten Kate, F. Barkhof, Adam J. Schwarz

**Affiliations:** 1 Department of Radiology & Nuclear Medicine, VU University Medical Center, Amsterdam UMC, Amsterdam, the Netherlands; 2 Queen Square Multiple Sclerosis Centre, Department of Neuroinflammation, UCL Queen Square Institute of Neurology, Faculty of Brain Sciences, University College London, London, UK; 3 Centre for Medical Image Computing (CMIC), Department of Computer Science, Faculty of Engineering Sciences, University College London, London, UK; 4 University College London, London, UK; 5 Takeda Pharmaceuticals Ltd., 40 Landsdowne St., Cambridge, MA 02139 USA; 6 Department of Radiology and Imaging Sciences, Indiana University School of Medicine, Indianapolis, IN USA

**Keywords:** Alzheimer’s disease, linical trials, atrophy, cognition

## Abstract

**Background:**

Longitudinal changes in volumetric MRI outcome measures have been shown to correlate well with longitudinal changes in clinical instruments and have been widely used as biomarker outcomes in clinical trials for Alzheimer’s disease (AD). While instances of discordant findings have been noted in some trials, especially the recent amyloid-removing therapies, the overall relationship between treatment effects on brain atrophy and clinical outcomes, and how it might depend on treatment target or mechanism, clinical instrument or imaging variable is not yet clear.

**Objective:**

To systematically assess the consistency and therapeutic class-dependence of treatment effects on clinical outcomes and on brain atrophy in published reports of clinical trials conducted in mild cognitive impairment (MCI) and/or AD.

**Design:**

Quantitative review of the published literature. The consistency of treatment effects on clinical and brain atrophy outcomes was assessed in terms of statistical agreement with hypothesized equal magnitude effects (e.g., 30% slowing of both) and nominal directional concordance, as a function of therapeutic class.

**Setting:**

Interventional randomized clinical trials.

**Participants:**

MCI or AD trial participants.

**Intervention:**

Treatments included were those that involved ingestion or injection of a putatively active substance into the body, encompassing both pharmacological and controlled dietary interventions.

**Measurements:**

Each trial included in the analysis reported at least one of the required clinical outcomes (ADAS-Cog, CDR-SB or MMSE) and at least one of the required imaging outcomes (whole brain, ventricular or hippocampal volume).

**Results:**

Data from 35 trials, comprising 185 pairwise comparisons, were included. Overall, the 95% confidence bounds overlapped with the line of identity for 150/185 (81%) of the imaging-clinical variable pairs. The greatest proportion of outliers was found in trials of anti-amyloid antibodies that have been shown to dramatically reduce the level of PET-detectable amyloid plaques, for which only 13/33 (39%) of observations overlapped the identity line. A Deming regression calculated using all data points yielded a slope of 0.54, whereas if data points from the amyloid remover class were excluded, the Deming regression line had a slope of 0.92. Directional discordance of treatment effects was also most pronounced for the amyloid-removing class, and for comparisons involving ventricular volume.

**Conclusion:**

Our results provide a frame of reference for the interpretation of clinical and brain atrophy results from future clinical trials and highlight the importance of mechanism of action in the interpretation of imaging results.

**Electronic Supplementary Material:**

Supplementary material is available in the online version of this article at 10.14283/jpad.2023.92.

## Introduction

**B**rain atrophy is a cardinal feature of Alzheimer’s disease (AD) and can be readily assessed in vivo using volumetric MRI (vMRI) scans. AD is associated with increased global atrophy, measured by reduced whole brain volume (WBV) or enlarged ventricular volume (VV), as well as more anatomically-specific atrophy of the medial temporal lobes, evidenced by reduced hippocampal volume (HCV) (1). In both observational studies and clinical trials, longitudinal changes in vMRI outcome measures have been shown to correlate well with longitudinal changes in cognition and function (2–5). Accordingly, vMRI has been widely used as a secondary or exploratory outcome measure in clinical trials for AD (1), with the hypothesis that effective treatment will slow down the rate of brain parenchymal tissue loss as well as slowing down the rate of cognitive decline. In a drug development context, the potential utility of brain atrophy as a biomarker is twofold. First, it may provide biological evidence of a slowing of the disease process in support of an observed clinical effect. Second, since vMRI outcomes are typically less variable in longitudinal change than clinical outcomes, imaging measures are of interest in the context of smaller Phase 2 studies or interim analyses, to potentially provide a biomarker signal consistent with a treatment being clinically effective (i.e., a slowing of brain atrophy) in a smaller sample than that required to detect an effect on clinical outcomes.

Power analyses of biomarkers in the context of clinical trials often compare sample size calculations to those required for clinical outcome measures, for some assumed amount of slowing of the untreated rate of change (e.g., 30% or 50% slowing in the treatment arm, relative to the placebo arm) (6). Such sample size comparisons implicitly assume that a given intervention will slow the clinical effect and the biomarker by the same relative amount, with relative powering then driven by the variability in each variable. While this concept applies to many neurological disorders, in the case of AD there are a number of clinical trials, across a range of therapeutic targets and mechanisms of action, that have reported treatment effects on both clinical and brain atrophy outcomes. In some cases, especially the recent amyloid-removing antibodies, discordant findings have been noted. However, the emphasis has often been on the presence or absence of statistical significance on each measure independently, and a systematic picture of the overall relationship between the directions and magnitudes of treatment effects on these two types of outcome measures, and how it might depend on treatment mechanism, clinical instrument or imaging variable is not yet clear.

To provide a clearer picture of the relationship between treatment effects on imaging and clinical measures in interventional AD trials, we performed a systematic meta-analytic review of the published literature to assess the consistency of treatment effects on the most common measures of brain atrophy and those on the most common measures of clinical progression.

## Methods

### Selection criteria and search strategy

Randomized controlled trials in MCI or AD of at least 12 months’ duration, and total sample size >80, reporting trial outcomes on cognition and MRI measures, were considered eligible for our analysis. Both clinical dementia and prodromal (mild cognitive impairment; MCI) disease stages were included. Cognitive measures needed to include at least one of the following: Alzheimer’s Disease Assessment Scale-Cognitive Subscale (ADAS-Cog), Clinical Dementia Rating Sum of Boxes (CDR-SB), or Mini Mental State Examination (MMSE). MRI outcomes needed to include at least one of the following: WBV, HCV, or VV. No selection based on image processing steps or software was made. These cognitive and imaging measures were chosen based on their widespread use in clinical trials. Treatments included were those that involved ingestion or injection of a putatively active substance into the body, encompassing both pharmacological and controlled dietary interventions. Purely lifestyle interventions such as physical exercise or meditation, and cognitive therapy were excluded.

Eligible studies were selected searching the PubMed database up to December 2022. We used a combination of search terms for the disease (“Alzheimer”, “mild cognitive impairment”) and for clinical trials (“clinical trial”, “randomized trial”). Only articles published in English were included. The title and abstract of articles were independently screened by two authors (MTK, AJS) for eligibility. Full papers were examined if relevant information could not be ascertained from the abstracts. Additional trials were included based on the authors’ personal knowledge, and systematic reviews were evaluated for any additional references.

### Data extraction

Relevant data extraction was performed independently by two authors (MTK, AJS) and any discrepancies resolved by consensus. Some studies reported more than one variation of the ADAS-Cog measure, in which case the ADAS-Cog11 was used, as this was the variant used in the majority of trials. If outcome data were only presented in figures, a determination was made as to whether any graphical representation of the data in the publication was of sufficient quality to allow a reasonable approximation of the values to be extracted from visual inspection. This determination was made by consensus between MTK and AJS. If yes, each of these two authors independently determined the values and the mean between them was used for each variable.

As a measure of treatment effect in each variable, we calculated the fractional slowing in change from baseline in the active treatment group relative to the control group. If the fractional (or percent) slowing was reported in the paper, we used this measure; otherwise, we computed it based on the reported changes per arm. For consistency, all measures are reported such that a positive percent change represents an improvement in the treatment group compared to control (e.g., 0.3 equates to a 30% slowing), and negative fractional change represents a worsening in the treatment group compared to the control group (Supplemental Material, Figure S1). In order to avoid meaninglessly inflated values of apparent treatment effects due to very small denominator values, we excluded data points in which there was minimal clinical decline in the control group, defined as less than +2 points on ADAS-Cog, −2 points on MMSE, or +1 points on CDR-SB. Three studies (7–9) were excluded based on this criterion; all three were performed in MCI populations without an amyloid inclusion requirement.

Each investigational agent was designated to a therapeutic class and target mechanism of action. For trials that reported results from more than one dose or more than one time point, the highest dose and/or last time point was designated as the primary readout for analysis.

### Data analysis

The primary data for analysis were the reported point estimates of fractional slowing in each pair of imaging and clinical outcomes. The reported 95% confidence intervals (95% CIs) of the point estimates of each variable were used as estimates of uncertainty in each variable. When not reported directly, the 95% CI values were derived from the reported statistical results. The 95% CIs were used because these reflect all sources of variability and limitations on study power, including sample size and length of follow-up. This approach also intrinsically accommodates scenarios where different sample sizes were available for different variables (e.g., due to imaging sub-studies or missing data) or not clearly reported in the source publications, and for the use of different statistical models in the analyses of the different studies.

The data were analysed in four ways. First, we assessed nominal concordance in the directionality of treatment effect on imaging and clinical variables both for all data points in the primary analysis. Data points with values of zero in one variable were determined as discordant. Second, for each data point (imaging-clinical variable pair) we tested the hypothesis that the treatment effects on each variable were consistent with an hypothesized equal-magnitude treatment effect on both imaging and clinical variables. A statistical distance from the identity line was used to assess how close each data point was to the identity line, which corresponds to equal-magnitude effects. This distance, *d*_*s*_, was calculated as a multiple of the distance from the data point to the point on the edge of the 95% CI ellipse corresponding to the bivariate distribution of the two variables that represents the minimum (or maximum, if the point is below the identity line) when rotated 45° so that the identity line coincides with the x-axis (Supplemental Material, Figure S2). This statistical distance can thus be thought of as a multiple of the 95% CI (so *d*_s_ < 1 means that the 95%CI ellipse overlaps the identity line). Other things being equal, smaller trials and/or shorter follow-up times result in larger confidence intervals and smaller weighting factors. For visualization purposes, we also calculated weighting factors for each data point as the inverse of the area of the ellipse whose major and minor axes are equal to twice the 95%CI of each variable (Supplemental Material, Figure S2), consistent with standard power calculations in which the test statistic is inversely proportional to the standard error of the dependent variable (which is related to the 95% CI by the T statistic for the relevant sample size, being 1.96–1.99 for the comparisons in this analysis). Third, we assessed the dependence of *d*_*s*_ on therapeutic class, imaging variable and clinical variable using ANOVA and post-hoc Tukey tests. Fourth, we performed a Deming regression to assess the slope of an assumed linear relationship between the magnitude of the treatment effect on the imaging and clinical variables. Unlike typical linear regression approaches, this method accounts for errors in both x and y variables in calculating the regression line. Statistical analyses were performed in R v4.2.1.

## Results

### Study selection

The primary search yielded 3410 records. Another 5 records were identified from other sources. Based on screening of titles and abstracts, 3271 records were excluded. 144 full-text articles were assessed for eligibility, from which 111 were excluded for various reasons (Supplemental Material, Figure S3). For some trials, the cognitive and imaging data were reported in separate papers, and some papers reported data from several trials. In total, we included data from 35 trials, and 26 different compounds (Table 1). The reported outcome measures varied across trials; ADAS-Cog was reported in 33, MMSE in 20, CDR-SB in 28, WBV in 25, VV in 27, and HCV in 27. Overall, 185 data points (unique pairs of clinical and imaging variables) were available for the analysis.

The start dates of the trials ranged from 1999 to 2017 (median 2012). 13 of the selected trials were designated as Phase 2 in the source reports (of which 4 were denoted as Phase 2a and 1 as Phase 2b), 2 as Phase 2/3 and 18 as Phase 3. Two of the trials did not specify a specific clinical phase. Four trials were conducted in an MCI population, 23 in AD, and 8 in a combination of MCI and AD participants (“Early AD”).

The majority of treatments evaluated (25/35 or 71% of the trials) targeted amyloid-related mechanisms. Of these, 9/35 (26%) trials evaluated large molecules, another 4/35 (11%) large molecules with the property of substantially removing amyloid plaques, 10/35 (29%) small molecules, and 2/35 (6%) active vaccines. A further 4/35 (11%) trials evaluated 3 different anti-tau molecules (a small molecule, a large molecule, and a vaccine), and 6/35 (17%) evaluated a variety of other mechanisms. The large molecule amyloid class included monoclonal antibodies targeting N-terminal fragment of or soluble amyloid protein, and plasma-derived polyclonal antibodies. The small molecule amyloid class included aggregation inhibitors, β-site APP cleaving enzyme (BACE) inhibitors, gamma secretase inhibitors, kinase inhibitors and a receptor for advanced glycation end products (RAGE) inhibitor. The “other” class included an acetylcholine esterase (AChE) inhibitor, insulin, an angiotensin II antagonist, a multinutrient formula, omega-3 fatty acid supplement and a compound with mixed mechanisms targeting synaptic plasticity and neuroprotection (Table 1).

**Table 1:** List of included trials, grouped by therapeutic class and target mechanism of action

**Trial number**	**Reference(s)**	**Compound**	**Trial name**	**NCT/other identifier**	**Year trial started**	**Phase**	**Class**	**Target MOA**	**Population**	**Measures**
1	(15, 16)	bapineuzumab	301 (non-carriers)	NCT00574132	2007	3	Amyloid, LM	mAb, N-terminal	AD	A,M,C; W,H,V
2	(15, 16)	bapineuzumab	302 (carriers)	NCT00575055	2007	3	Amyloid, LM	mAb, N-terminal	AD	A,M,C; W,H,V
3	(17)	bapineuzumab	3001 (carriers)	NCT00676143	2008	3	Amyloid, LM	mAb, N-terminal	AD	A,C; W
4	(17)	bapineuzumab	3000 (non-carriers)	NCT00667810	2009	3	Amyloid, LM	mAb, N-terminal	AD	A,C; W
5	(18)	crenezumab	CREAD	2015-003034-27	2016	3	Amyloid, LM	mAb, oligomeric	MCI+AD	A,M,C; W,H,V
6	(19]	crenezumab	ABBY	NCT01343966	2011	2	Amyloid, LM	mAb, soluble amyloid	AD	A,C; W,H,V
7	(20)	solan ezumab	EXPEDITION!&2 (mild)	NCT00905372, NCT00904683	2009	3	Amyloid, LM	mAb, soluble amyloid	AD	A,M,C; W/V
8	(21)	solan ezumab	EXPEDITION3	NCT01900665	2013	3	Amyloid, LM	mAb, soluble amyloid	AD	A,M,C; W/V
9	(22)	IVIg / Gammagard	ns	NCT00818662	2008	3	Amyloid, LM	pAb, plasma-derived	AD	A; W,H,V
10	(23)	aducanumab	EMERGE	NCT02484547	2015	3	Amyloid, LM, amyloid remover	mAb, plaque reducer	MCI+AD	A,M,C; W,H,V
11	(23)	aducanumab	ENGAGE	NCT02477800	2015	3	Amyloid, LM, amyloid remover	mAb, plaque reducer	MCI+AD	A,M,C; W,H,V
12	(24)	donanemab	TRAILBLAZER-ALZ	NCT03367403	2017	2	Amyloid, LM, amyloid remover	mAb, plaque reducer	MCI+AD	A,M,C; W,H,V
13	(25)	lecanemab	BAN2401-G000-201	NCT01767311	2012	2b	Amyloid, LM, amyloid remover	mAb, plaque reducer	MCI+AD	A,C; W,H,V
14	(26)	scyllo-inositol	ns	NCT00568776	2007	2	Amyloid, SM	Aggregation inhibitor	AD	A,M,C; V
15	(27)	tramiprosate	Alphase	ns	2004	3	Amyloid, SM	Aggregation inhibitor	AD	A,C;H
16	(28, 29)	lanabecestat	AMARANTH	NCT02245737	2014	2/3	Amyloid, SM	BACE inhibitor	MCI+AD	A,C; W,H,V
17	(28, 29)	lanabecestat	DAYBREAK-ALZ	NCT02783573	2016	3	Amyloid, SM	BACE inhibitor	AD	A,C; W,H,V
18	(30)	verubecestat	AD	NCT01739348	2012	3	Amyloid, SM	BACE inhibitor	AD	A,M,C; H
19	(31)	verubecestat	MCI	NCT01953601	2013	3	Amyloid, SM	BACE inhibitor	MCI	A,M,C; H
20	(32)	avagacestat	ns	NCT00890890	2009	2	Amyloid, SM	Gamma secretase inhibitor	MCI	A,M,C; W,H,V
21	(3)	semagacestat	IDENTITY1&2	NCT00594568	2008	3	Amyloid, SM	Gamma secretase inhibitor	AD	A,M,C; H,V
22	(33)	AZD0530	ns	NCT02167256	2014	2a	Amyloid, SM	Kinase inhibitor	AD	A,M,C; W,H,V
23	(34)	PF-04494700	ns	NCT00566397	2007	2	Amyloid, SM	RAGE inhibitor	AD	A,C;H
24	(35, 36)	AN1792	ns	ns	2001	2a	Amyloid, vaccine	Active vaccine	AD	A; W,H,V
25	(37)	vanutide cridificar	ns	NCT00960531, NCT00955409	2009	2a	Amyloid, vaccine	Active vaccine	AD	A,M,C; W,H,V
26	(38)	losartan	RADAR	2012-003641-15	2014	2	Other	Angiotensin II.1 antagonist	AD	A,M; W
27	(39)	intranasal insulin	ns	NCT01767909	2014	2/3	Other	Insulin	MCI+AD	A,C;H
28	(40)	fortasyn connect / Souvenaid	LipiDiDiet	NTR1705 (NL)	2009	ns	Other	Multinutrient	MCI	C; W,H,V
29	(41])	Docosahexaenoic acid (DHA)	ns	NCT00440050	2007	ns	Other	Omega-3 fatty acid supplement	AD	A,M,C; W,H,V
30	(42)	rivastigmine	InDDEx	NCT00000174	1999	3	Other	AChE inhibitor	MCI	A; V
31	(43)	Edonerpic maleate	ns	ns	2014	2a	Other	Mixed mechanisms	AD	A,M; W,H,V
32	(44)	semorinemab	Tauriel	NCT03289143	2017	2	Tau, LM	mAb	MCI+AD	A,C; W,H,V
33	(45)	LMTM	TRx-237-005	NCT01689233	2012	3	Tau, SM	Aggregation inhibitor	AD	A,M; W,H,V
34	(46)	LMTM	ns	NCT01689246	2013	3	Tau, SM	Aggregation inhibitor	AD	A,M; V
35	(47)	AADvacl	ADAMANT	2015-000630-30	2016	2	Tau, Vaccine	Active vaccine	AD	C; H,V

A = ADAS-Cog; AChE = acetylcholinesterase; AD = Alzheimer’s disease dementia; BACE = beta-secretase; C = CDR-SB; H = hippocampal volume; LM = large molecule; M = MMSE; mAb = monoclonal antibody; MAO = monoamine oxidase; MCI = mild cognitive impairment; MOA = mechanism of action; NMDAR = N-methyl-D-aspartate receptor; ns = not specified; pAb = polyclonal antibody; RAGE = receptor for advanced glycation end products; SM = small molecule; V = ventricular volume; W = whole brain volume.

### Relationships between treatment effects on brain volumes and cognition

**Figure 1:**
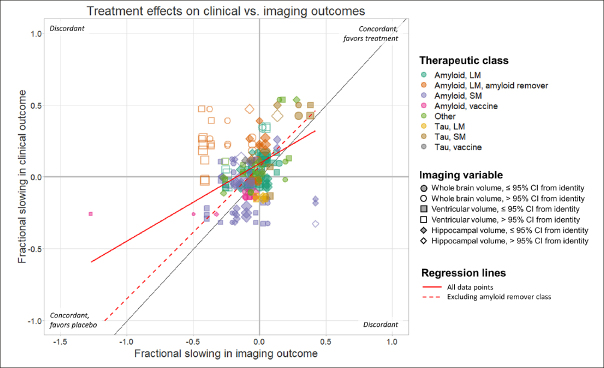
Scatter plot showing the relationship between published treatment effects on clinical and imaging outcomes for all comparisons in the analysis, expressed as fractional slowing in the treatment arm relative to the control arm

**Figure 2:**
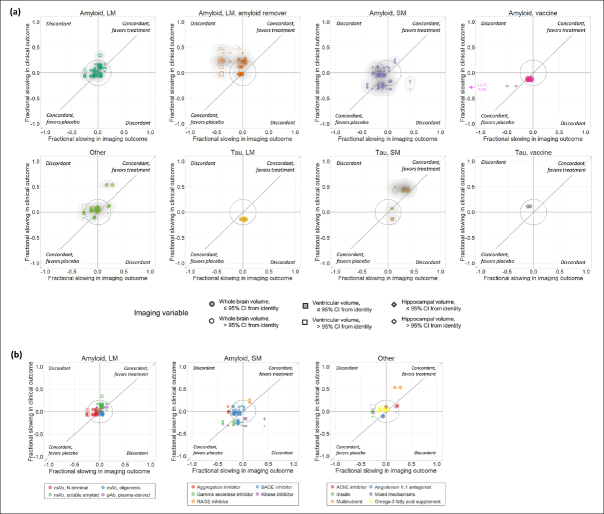
(a) Scatter plots of treatment effects on clinical vs. imaging outcomes for each therapeutic class independently. (b) For those classes comprising diverse mechanisms of action (amyloid large molecule, amyloid small molecule, and other), data points within each therapeutic class are coloured according to the specific mechanism of action

The overall relationship between the point estimates of treatment effects on all pairs of imaging and clinical outcomes is illustrated in Figure 1. Overall, 102/185 (55%) of these were concordant (directionally consistent for imaging and clinical variables). Differences between therapeutic classes in the pattern of relationships and degree of concordance can be appreciated when plotted separately (Figure 2). For specific classes, concordance was 30/52 (58%) for large molecule amyloid, 28/59 (57%) for small molecule amyloid, 12/12 (100%) for amyloid vaccines, 7/8 (88%) for tau small molecule, 2/6 (33%) for tau large molecule, 0/2 (0%) for tau vaccine and 12/24 (50%) for the “other” category. In contrast, concordance was 9/33 (27%) for large molecule amyloid removers, a class that visually appears as the most clearly discordant in Figures 1 & 2. For all classes combined except the large molecule amyloid remover class, 93/152 (61%) of the data points were directionally concordant. However, many data points were close to the origin, corresponding to small treatment effects. If only data points further than 0.25 from the origin were considered, reflecting treatment effects more likely to be clinically meaningful, then 39/49 (80%) of the data points from therapeutic classes other than the amyloid removers were directionally concordant, compared with just 3/21 (14%) of the data points from the amyloid removers class (see Supplemental Material).

For example, a value of 0.5 corresponds to a 50% lower change in the treatment arm, whereas a value or −0.3 corresponds to a 30% faster decline in the treatment arm. Each data point represents a comparison between the effect on one clinical instrument and one imaging outcome. Data points are coloured according to therapeutic class and sized in inverse proportion to the product of the 95% CIs of the point estimates. Filled symbols indicate data points whose 95% CIs overlap with the identity line. Deming regression lines are shown for all data points (solid line) and for data points other than those in the amyloid remover class (dashed line).

Filled symbols indicate data points whose 95% CIs overlap with the identity line.

**Figure 3:**
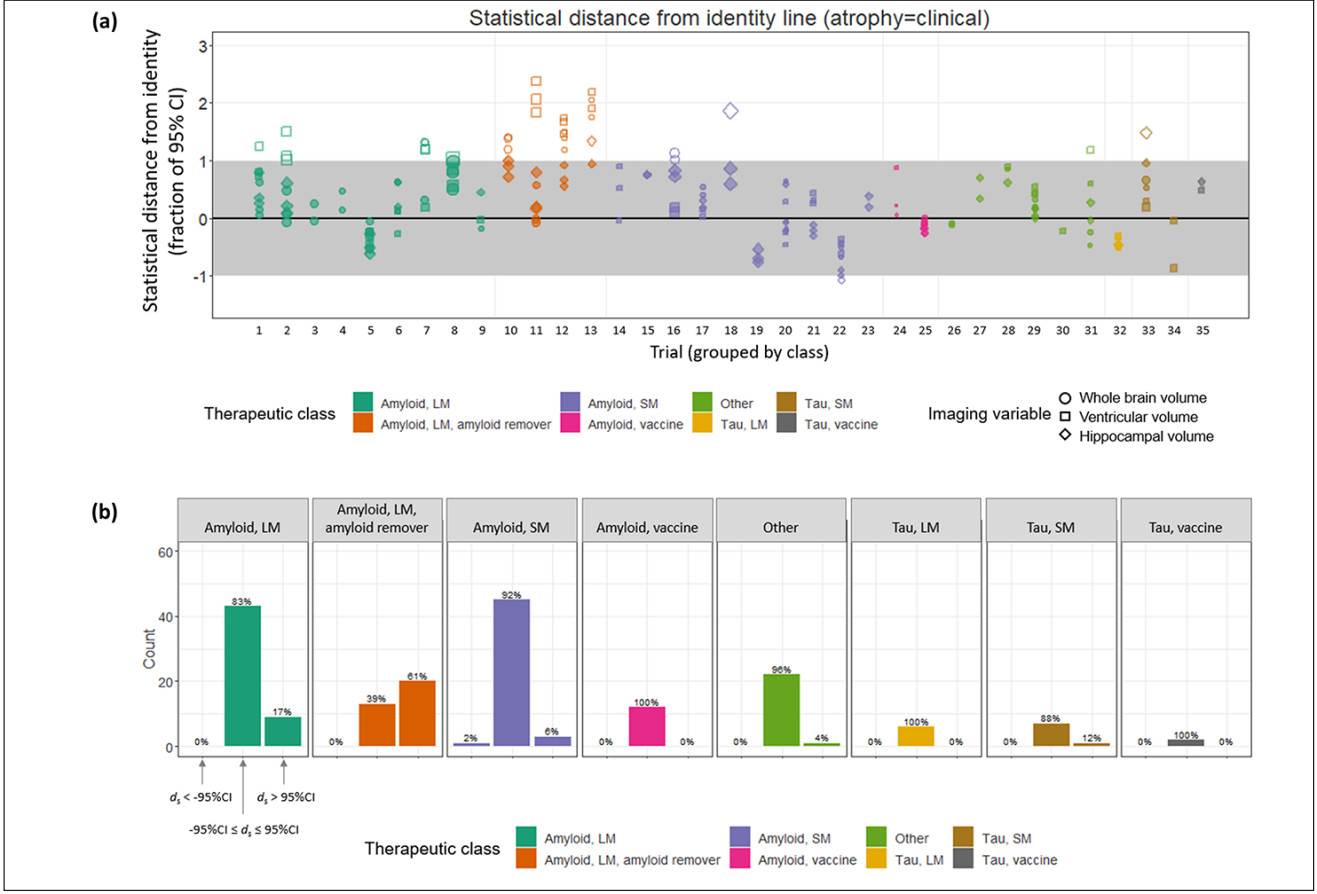
(a) Each data point, grouped by trial and therapeutic class, is plotted as its statistical distance from the line of identity. Trial numbers correspond to those in Table 1. Filled symbols indicate data points whose 95% CIs overlap with the identity line. (b) Within each therapeutic class, histograms showing the proportion of data points whose 95% CIs overlap the identity line, those non-overlapping above the identity line and those non-overlapping below the identity line

Directional concordance of treatment effects based on the point estimates does not take into account the statistical uncertainties around them. When these same data were considered in terms of their statistical distance *d*_*s*_ from the identity line, we found that, overall, the 95% CIs overlapped the identity line for 150/185 (81%) of the data points (Figure 3). That is, the point estimates of 81% of the sample were consistent, within measurement uncertainty, with equivalent magnitudes of treatment effects on clinical and imaging outcomes. Of the outlier data points, 34/185 (18%) were >95% CI above the identity line, whereas only 1/185 was >95% CI below the identity line. The greatest number of outliers was present for the amyloid remover class, for which only 13/33 (39%) of observations overlapped the identity line. These outliers included all (11/11) of the data points involving ventricular volume, 8/11 of those involving whole brain volume but only 1/11 of those involving hippocampal volume. Across the other therapeutic classes, 137/152 (90%) of the observations were consistent with the equivalent magnitude hypothesis. The fifteen outliers for the non-amyloid-removing therapies arose from large molecule amyloid trials in 9/15 (60%) cases, small molecule amyloid in 4/15 cases, along with one from a small molecule tau trial and one from the “other” category. The variable pairs corresponding to these outliers included ventricular volume in 8/15 (53%) of cases, whole brain volume in 4/15 (27%) of cases and hippocampal volume in 3/15 (20% of cases). In contrast, the three clinical variables were represented more evenly; 5/15 ADAS-Cog, 6/15 MMSE and 4/15 CDR-SB (Supplemental Material, Table S1).

In an ANOVA analysis, the statistical distance was strongly dependent on therapeutic class (F = 17.2, p ∼ 10^−16^) and on the imaging variable (F = 5.8, p < 0.005), but not on the clinical variable (F = 0.72, p = 0.5). It can be observed in Figure 3 that this effect is primarily driven by the amyloid remover class, which in post-hoc Tukey comparisons exhibited a significantly greater average *d*_*s*_ compared with every other class except the tau vaccine class (p_adj_ = 0.001 vs. Tau SM class; p_adj_=0.56 vs. tau vaccine class; p < 10^−7^ vs. all other classes). The only other significant difference in *d*_*s*_ between classes was between the Tau LM and Amyloid LM classes (p_adj_<0.05). The difference in *d*_*s*_ across imaging variables was driven by VV whose values were on average greater than both WBV (p_adj_<0.05) and HCV (p_adj_<0.005). There was an overall bias toward positive *d*_s_ values, indicating a trend toward stronger beneficial treatment effects on clinical compared with imaging variables.

A Deming regression calculated using all data points yielded a slope of 0.54. If data points from the amyloid remover class were excluded, the Deming regression line had a slope of 0.92 (Figure 1).

## Discussion

This systematic survey of the relationships between treatment effects on brain atrophy and clinical instruments in MCI/AD trials revealed a high statistical consistency between the two types of outcome, despite a lower nominal concordance in the directionality of the effects. For the majority (81%) of all comparisons, and an even higher fraction (90%) of comparisons from non-amyloid-removing interventions, the effects of the interventions on brain atrophy and clinical outcomes were consistent within statistical expectations (defined as the 95% CI bounds of uncertainty on each variable). Concordance rates were lower, with 55% overall and 61% of the data points from non-amyloid-removing interventions being directionally concordant. This lower nominal concordance likely reflects in part the fact that most of the treatment effect magnitudes –- on one or both variables –- were relatively modest and not themselves statistically significant, as concordance for non-amyloid-removing interventions increased to 80% if only data points greater than 0.25 from the origin were considered. Directional discordance may not be reliable in the case where overall effect magnitudes are small and likely below that for which the study was powered. This situation is exacerbated in smaller (e.g., Phase 2) studies for which the residual variability around the point estimates is greater.

The class of amyloid removing therapies behaved qualitatively differently and showed the strongest evidence of discordance between treatment effects on brain atrophy and clinical outcomes, with an overall pattern of a slowing of clinical decline being accompanied by accelerated brain volume loss. Specifically, and in contrast to the behaviour observed for the other classes (above), only 39% of the data points were statistically consistent with equivalent effects on imaging and clinical outcomes, and only 27% were nominally directionally concordant. The slope of the regression line through all data points was 0.92 (close to the hypothesized value of 1) if the amyloid removing class was excluded, compared with 0.54 if points from that class were included. Interestingly, this discordant behaviour was most pronounced for effects on ventricular volume. None (0/11) of the data points involving ventricular volume from this class (across three molecules and four separate trials) had 95% CIs that overlapped the identity line, in contrast to 3/11 of those involving whole brain volume and 10/11 of those involving hippocampal volume. This suggests that the accelerated brain volume loss observed for this class may be driven by volume reductions in white matter, and is consistent with a recent meta-analysis that also found a significant overall acceleration of ventricular enlargement for amyloid-removing therapies, associated with ARIA prevalence (10). However, whole brain volume and ventricular enlargement are also anatomically non-specific, in contrast to the hippocampus which is more indicative of AD-related neurodegeneration.

The question of what is driving this phenomenon is an important one, especially whether it represents accelerated neurodegeneration, or treatment-induced changes in non-neurodegenerative inflammatory or other processes affecting bulk tissue volumes (e.g., edema, hydration, CSF fluid shifts), similar to the short-term “pseudo-atrophy” that has been described with disease-modifying therapies in Multiple Sclerosis (11, 12). More targeted molecular imaging studies to elucidate contributions of inflammatory changes would be insightful. Additional analyses of the vMRI data from those trials, including a separate quantitation of white matter volume per se and more granular grey matter volumes (e.g., regional cortical volumes or thinning, especially those associated with AD-related neurodegeneration to greater and lesser degrees), would also shed additional light on these observations, and may help explain the sometimes discrepant results across vMRI variables observed in the same trial. For example, it would be interesting to know whether the regional patterns of brain volume changes are proportional to regional changes in the placebo arm (providing an indication of whether the treatment effects are suggestive of a change in disease progression, or rather indicate a non-specific effect (13)). It would also be enlightening to know whether there is a relationship between regional pattern of brain volume changes, and the anatomical distribution of amyloid (shown to be the case in a recent analysis of the verubecestat trial in mild to moderate AD (14)), or the anatomical pattern of amyloid removal. Of note, the verubecestat trial also contained an early (3 month) time point, demonstrating that the accelerated volume loss was achieved within the first 3 months and was hence a relatively acute effect of the intervention; from 3–18 months the slopes of volume changes in the active treatment arms were parallel to those in the placebo arm (14). In contrast, the published data for the amyloid-remover class compounds indicate an ongoing acceleration of brain tissue loss rather than an acute effect.

Interestingly, we also noted that the class of anti-amyloid large molecules without notable amyloid-removing properties (as measured by amyloid PET) and the small-molecule amyloid class each exhibited a slightly skewed relationship intermediate between the patterns observed for the amyloid-removing therapies and those of the other categories, consistent with the notion that these other anti-amyloid mechanisms may to a lesser degree be related to the discordance and emergence of the increased volume loss associated with the amyloid remover class. Of note, a temporospatial analysis of the accelerated brain volume loss elicited by the BACE inhibitor verubecestat using a more granular parcellation of the brain (14) revealed that those effects were primarily acute in nature (stabilizing after 3 months) and more pronounced in amyloid-rich regions of the cortex. While similar fine-grained regional analyses of the vMRI results from the amyloid removing antibody trials have not been published, the temporal profiles of treatment effects on the atrophy outcomes assessed in this paper appear to show a more progressive rather than acute effect. Thus, different anti-amyloid mechanisms of action may result in different manifestations of increased brain volume loss.

The present findings also serve as a reminder that even for nominally well-powered trials (typically at 80% or 90% power, with a 5% significance level), uncertainty in the results remains, as evidenced by the scatter of trial data points around the identity line and the confidence intervals shown in Supplemental Figures S5–S13. Moreover, the different variables have different levels of intrinsic variability, with effects on cognitive outcomes typically having larger confidence intervals than imaging outcomes, for similar sample sizes. Smaller, phase 2, trials may be nominally powered for imaging but not clinical outcomes. We focused our analysis on imaging and clinical outcomes that were most commonly reported and, in the case of ADAS-Cog or CDR-SB especially, often the primary outcome of the trial overall. While not all trials reported findings on all these variables, this choice provided a strong basis for comparison across the many trials in our analysis.

These findings provide an historical context of treatment effects upon which new findings may be compared and to ascertain whether newly observed effects are within the historical range of variability. Plotting the active vs. placebo differences on clinical and imaging variables simultaneously, along with the uncertainties in these point estimates, is a straightforward way of determining if new findings are consistent within statistical error, and how they compare to those from previous trials and different mechanisms of action.

We note several limitations of this study. First, the analyses are based on reported outcome data at trial level, and not on individual subject-level data. Second, not all papers presented the required tabulated data and thus, for some studies, data values were extracted visually from figures. To minimize bias and error, these values were estimated independently and carefully by two authors and the average was used in the analysis. Third, there were some differences across trials in the analytical techniques and software tools to determine atrophy measures, although the measures used were consistent within trials for treatment and placebo groups. Fourth, we attempted to group compounds into classes based on their reported mechanism of action, but due to the great diversity reported, some mechanisms are as yet poorly represented; the greatest diversity of compounds and trials was available for the various anti-amyloid classes. Fifth, our basic hypothesis was that the magnitude of the treatment effect (expressed as percent slowing of decline) was the same for clinical and imaging outcomes. This contains two assumptions: first, that the relative treatment effects themselves are equivalent in magnitude and, second, that this relationship is linear and consistent across the range of effect sizes and variable values observed. The volumetric outcomes are fully quantitative and have been found to be linear across a range of values and disease stages, whereas the cognitive outcomes are discrete metrics based on granular test scoring, with well-defined floor and ceiling values. The relationships may thus be non-linear, although we found no strong indication of that, within statistical uncertainty, in our analysis.

In conclusion, in this meta-analytic review of published AD clinical trial results up to December 2022, we found evidence supporting a consistent relationship, within expected statistical uncertainty, between treatment effects on brain atrophy measures and those on standard instruments of global cognition for most therapeutic mechanisms of action. However, certain therapeutic mechanisms –- especially those with strong amyloid removing properties –- give rise to a decoupling of the otherwise strong relationships between atrophy and cognition observed in natural history studies and trial placebo arms, and lead to an apparent acceleration of brain atrophy. The accelerated brain tissue loss associated with amyloid removal was preferentially associated with ventricular expansion and may be primarily mediated by volume changes in the white matter. Directional concordance between effects on atrophy and clinical outcomes is likely to be reliable only for larger magnitudes of treatment effect. Overall, our findings provide a concise summary of relative treatment effects on clinical and brain atrophy outcomes that may be a useful reference framework for the interpretation of future AD trial results.

## Supplemental Material

Appendix

## Abbreviations

AD: Alzheimer’s disease
ADAS-Cog: Alzheimer’s Disease Assessment Scale-Cognitive Subscale
CDR-SB: Clinical Dementia Rating scale Sum of Boxes
HCV: hippocampal volume
MCI: mild cognitive impairment
MMSE: Mini Mental State Examination
vMRI: volumetric magnetic resonance imaging
VV: ventricular volume
WBV: whole brain volume.
