# Supplementary material for: Consistency between Treatment Effects on Clinical and Brain Atrophy Outcomes in Alzheimer’s Disease Trials
Source: J Prev Alzheimers Dis. 2023 Jul 13;11(1):38–47. doi: 10.14283/jpad.2023.92 (PMC10994869; doi:10.14283/jpad.2023.92)
Supplement: Supplementary file 1 — Appendix [file 42414_2023_92_MOESM1_ESM.pdf]

Supplemental Material

Supplemental Methods

Figure S1 shows the schematic axes on which the data points were plotted, and interpretation of each quadrant. **Figure S2** graphically illustrates the concept and construction of the “statistical distance” d_s, whereby the distance of each data point to the line of identity is expressed as a fraction of the 95% confidence intervals on each variable.

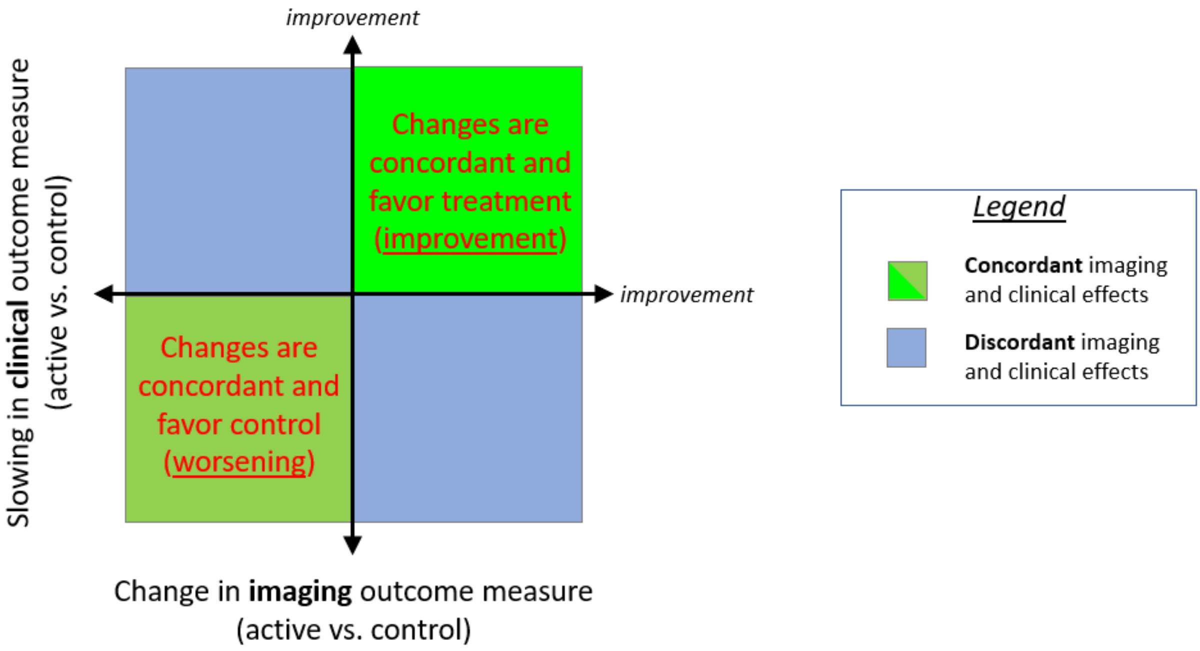

Figure S1: *Schematic diagram illustrating the directionality of the axes used to plot treatment effects on pairs of imaging and clinical variables (i.e., the result of the comparison between the active treatment arm and the control arm). For example, the top right quadrant corresponds to concordant effects on both variables, in a direction favouring the active treatment.*

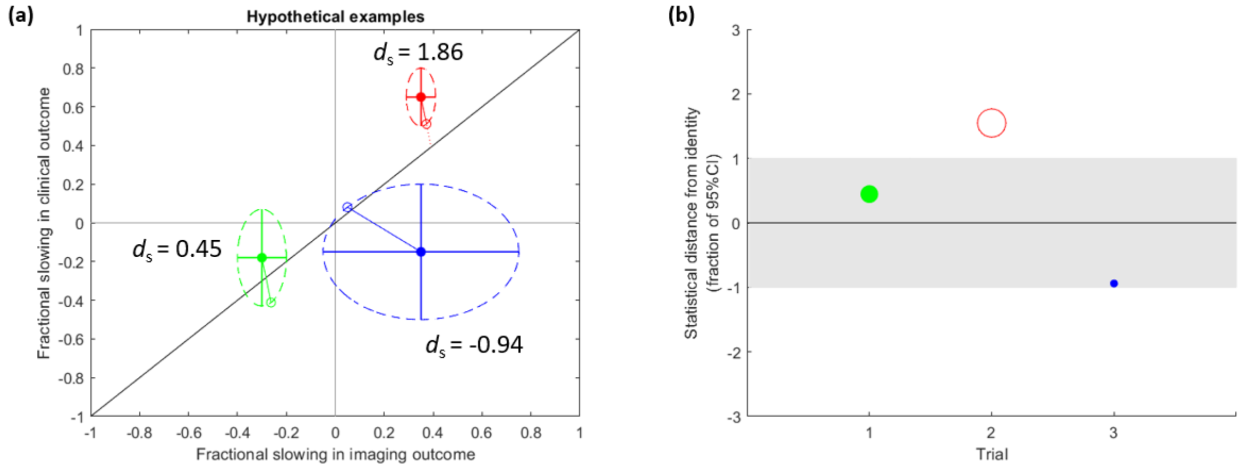

Figure S2: *Schematic diagrams illustrating statistical distance d_s from the line of identity. (a) Three hypothetical data points with error bars equal to the 95% CI in the point estimate of the treatment effect on the imaging outcome (x-axis) and clinical outcome (y-axis), with those error bars defining ellipses describing the 95% CIs of the bivariate distributions. The ellipses for the green and blue points overlap the identity line, whereas those for the red point do not. Points to the lower right of the identity line are signed negative. (b) Each data point plotted as "statistical distance" d_s (i.e., normalized to the 95% CI) for each trial in turn. The grey rectangle shows the range of $|d_s|$< 95% CI. The green and blue points lying within the range of 95% CI are filled, whereas the red point, lying outside this range, is empty. The size of the points is inversely proportional to the 95% CI ellipses. These plotting conventions are also employed for the data in the main body of the paper.*

Supplemental Results

Study selection

Figure S3 illustrates the study selection flow for the literature review performed in this study.

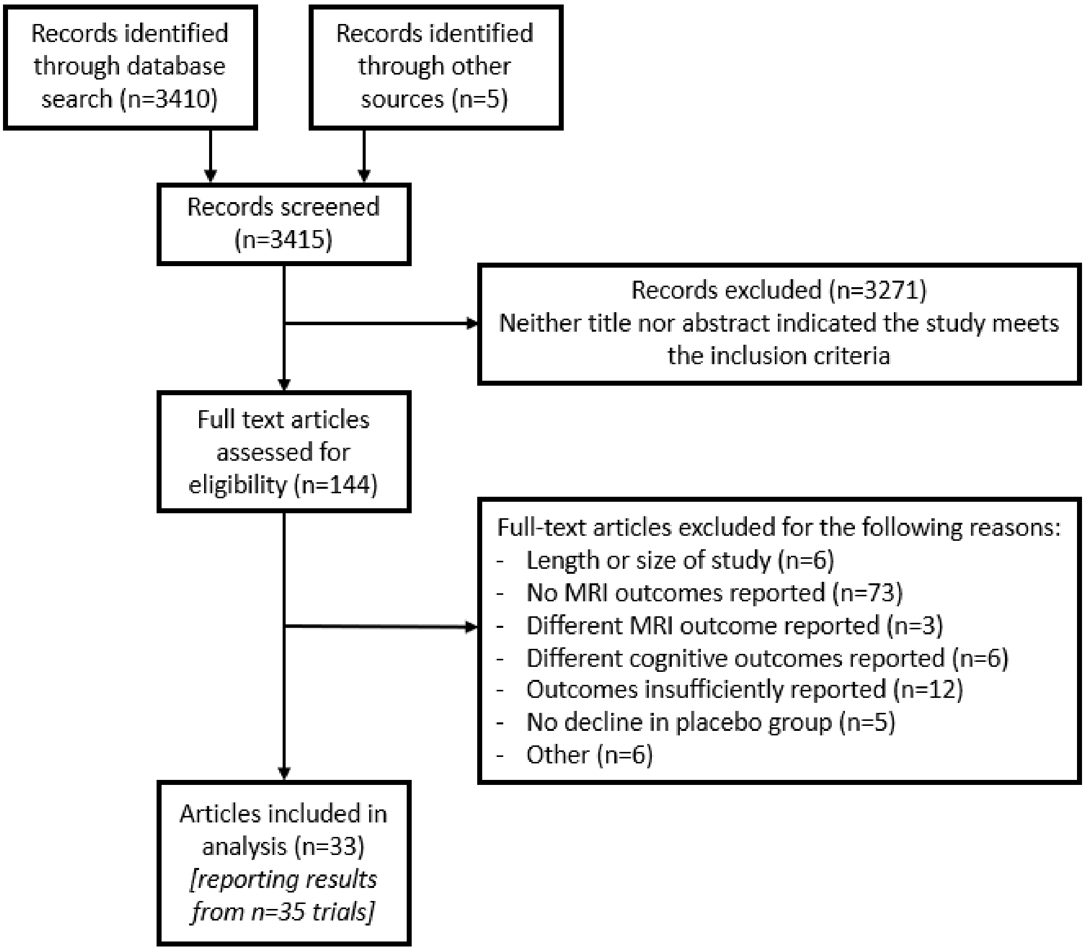

Figure S3: *Flow chart diagram of study selection for analysis. Note that some trial results were reported over two articles (one for clinical results, one for imaging results) and some articles reported results from two trials.*

Relationships between treatment effects on brain atrophy and cognition

Concordance (data points > 25% from origin)

We also investigated nominal directional concordance between treatment effects on clinical outcomes and brain atrophy when considering only those data points lying greater than 0.25 from the origin, corresponding to effects of more meaningful magnitude and more likely to be clinically significant. Overall, 42/70 (60%) were directionally concordant. For specific classes, concordance rates were 6/7 (86%) for large molecule amyloid, 17/25 (68%) for small molecule amyloid, 3/3 (100%) for amyloid vaccines, 6/6 (100%) for small molecule tau, and 9/14 (64%) for the "other" class. Again, the large molecule amyloid remover class was lower, with 3/21 (14%) of the data points further from the origin being concordant. For all classes combined except the large molecule amyloid remover class, 39/49 (80%) of the data points were directionally concordant.

Statistical outliers (d_s > 1)

Details of the outlier data points (i.e., those with d_s > 1, or whose 95%CIs do not overlap the line of identity) are provided in **Table S1**. Overall, there were 35/185 (19%) outliers. The majority (20/35) of these were found in the amyloid remover class, for which 20/33 (61%) were outliers.

Table S1: *Data points not statistically consistent with equal magnitude effects on clinical and imaging (brain atrophy) variables*

Trial number	Compound	Trial Name	Population	Class	Imaging variable	Clinical variable
**1**	bapineuzumab	301 (non-carriers)	AD	Amyloid, LM	VV	MMSE
**2**	bapineuzumab	302 (carriers)	AD	Amyloid, LM	VV	ADASCOG
**2**	bapineuzumab	302 (carriers)	AD	Amyloid, LM	VV	CDRSB
**2**	bapineuzumab	302 (carriers)	AD	Amyloid, LM	VV	MMSE
**7**	solanezumab	EXPEDITION1&2 (mild)	AD	Amyloid, LM	WBV	ADASCOG
**7**	solanezumab	EXPEDITION1&2 (mild)	AD	Amyloid, LM	WBV	MMSE
**7**	solanezumab	EXPEDITION1&2 (mild)	AD	Amyloid, LM	VV	ADASCOG
**7**	solanezumab	EXPEDITION1&2 (mild)	AD	Amyloid, LM	VV	MMSE
**8**	solanezumab	EXPEDITION3	AD	Amyloid, LM	VV	CDRSB
**10**	aducanumab	EMERGE	MCI+AD	Amyloid, LM, amyloid remover	WBV	ADASCOG
**10**	aducanumab	EMERGE	MCI+AD	Amyloid, LM, amyloid remover	WBV	CDRSB
**10**	aducanumab	EMERGE	MCI+AD	Amyloid, LM, amyloid remover	WBV	MMSE

**10**	aducanumab	EMERGE	MCI+AD	Amyloid, LM, amyloid remover	VV	ADASCOG
**10**	aducanumab	EMERGE	MCI+AD	Amyloid, LM, amyloid remover	VV	CDRSB
**10**	aducanumab	EMERGE	MCI+AD	Amyloid, LM, amyloid remover	VV	MMSE
**11**	aducanumab	ENGAGE	MCI+AD	Amyloid, LM, amyloid remover	VV	ADASCOG
**11**	aducanumab	ENGAGE	MCI+AD	Amyloid, LM, amyloid remover	VV	CDRSB
**11**	aducanumab	ENGAGE	MCI+AD	Amyloid, LM, amyloid remover	VV	MMSE
**12**	donanemab	TRAILBLAZER-ALZ	MCI+AD	Amyloid, LM, amyloid remover	WBV	ADASCOG
**12**	donanemab	TRAILBLAZER-ALZ	MCI+AD	Amyloid, LM, amyloid remover	WBV	CDRSB
**12**	donanemab	TRAILBLAZER-ALZ	MCI+AD	Amyloid, LM, amyloid remover	WBV	MMSE
**12**	donanemab	TRAILBLAZER-ALZ	MCI+AD	Amyloid, LM, amyloid remover	VV	ADASCOG
**12**	donanemab	TRAILBLAZER-ALZ	MCI+AD	Amyloid, LM, amyloid remover	VV	CDRSB
**12**	donanemab	TRAILBLAZER-ALZ	MCI+AD	Amyloid, LM, amyloid remover	VV	MMSE
**13**	lecanemab	BAN2401-G000-201	MCI+AD	Amyloid, LM, amyloid remover	WBV	ADASCOG
**13**	lecanemab	BAN2401-G000-201	MCI+AD	Amyloid, LM, amyloid remover	WBV	CDRSB
**13**	lecanemab	BAN2401-G000-201	MCI+AD	Amyloid, LM, amyloid remover	VV	ADASCOG
**13**	lecanemab	BAN2401-G000-201	MCI+AD	Amyloid, LM, amyloid remover	VV	CDRSB
**13**	lecanemab	BAN2401-G000-201	MCI+AD	Amyloid, LM, amyloid remover	HCV	ADASCOG
**16**	lanabecestat	AMARANTH	MCI+AD	Amyloid, SM	WBV	ADASCOG
**16**	lanabecestat	AMARANTH	MCI+AD	Amyloid, SM	WBV	CDRSB
**18**	verubecestat	AD	AD	Amyloid, SM	HCV	MMSE
**22**	AZD0530	-	AD	Amyloid, SM	HCV	CDRSB
**31**	Edonerpic maleate/T-817MA	-	AD	Other	VV	ADASCOG
**33**	LMTM	TRx-237-005	AD	Tau, SM	HCV	MMSE

Distribution of d$_s$ *values as a function of therapeutic class*

As a supplement to Figure 3 in the main paper, **Figure S4** shows density plots of the values of d_s for each therapeutic class. The curve for the amyloid remover class is clearly right-shifted relative to the other classes, which are predominantly within $|d_s|<1$.

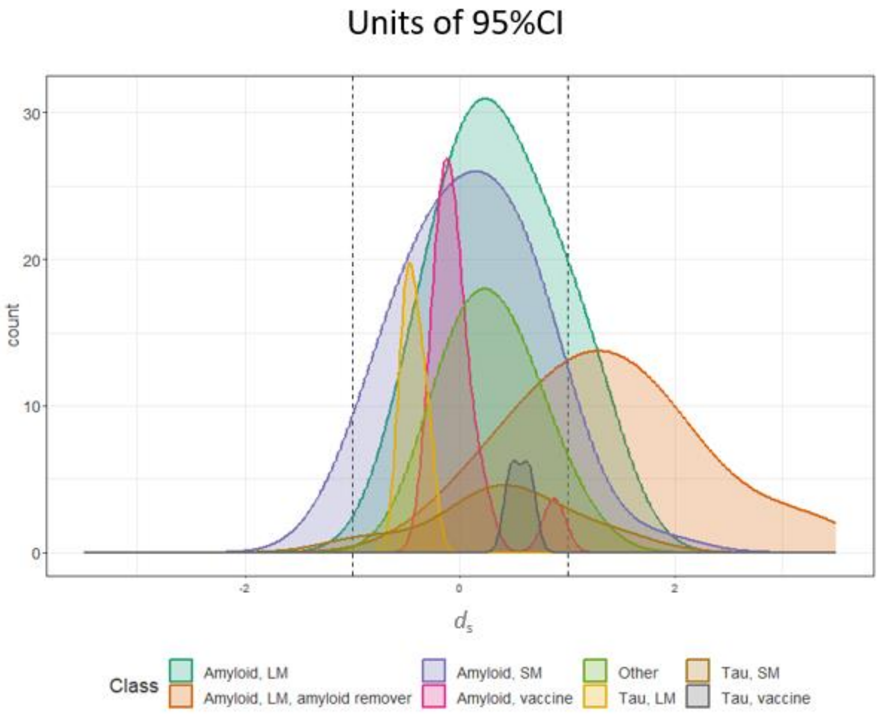

Figure S4: *Density plots of the distribution of d_s within each of the therapeutic classes. The vertical dashed lines indicate $d_s=\pm1$, so that values within the dashed lines correspond to points whose 95% CIs overlap the line of identity.*

Plots of individual clinical and imaging variable pairs

Figures S5-S13 depict the data points for each pair of clinical and imaging variables individually. The data are represented by the 95% CI ellipses (cf. Figure S2) with the point estimate depicted as the trial number corresponding to Table 1 in the main paper.

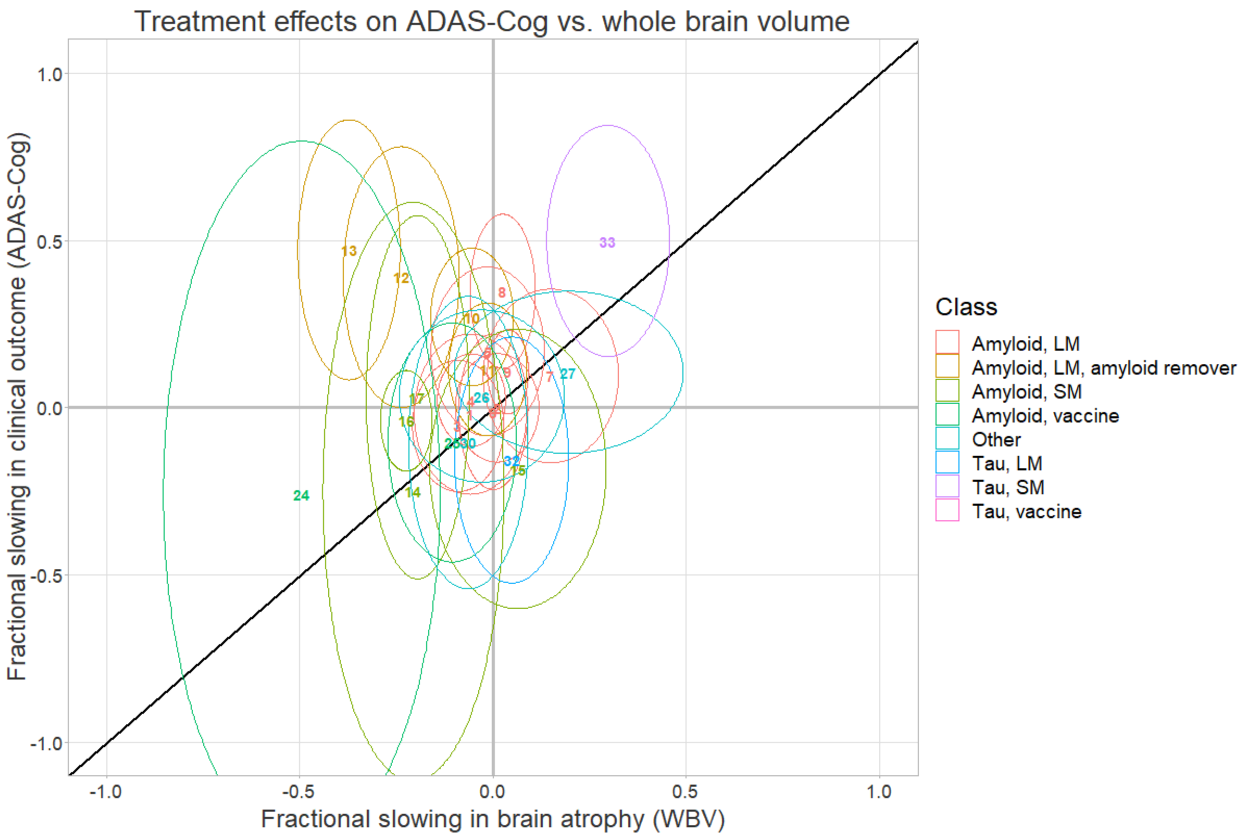

Figure S5: *Scatter plot of treatment effects on data points corresponding to WBV vs. ADAS-Cog.*

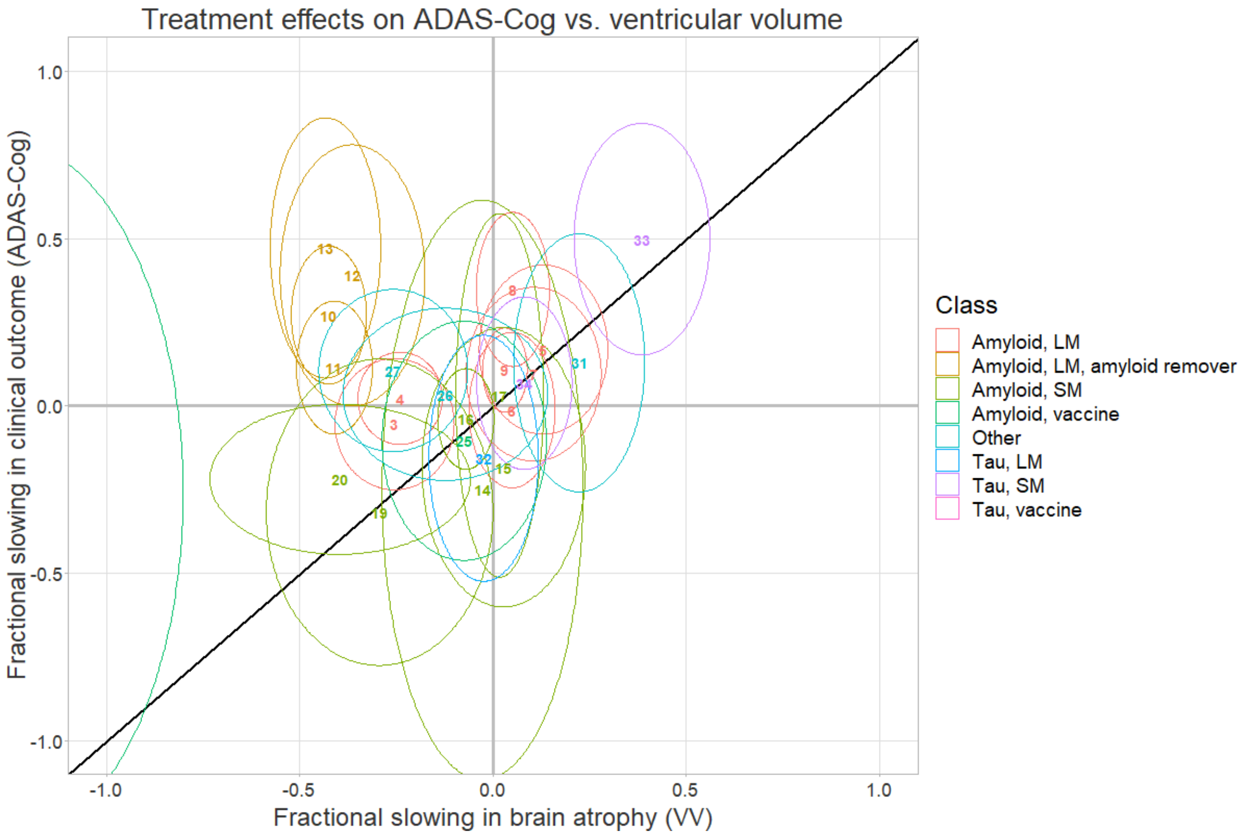

Figure S6: *Scatter plot of treatment effects on data points corresponding to VV vs. ADAS-Cog.*

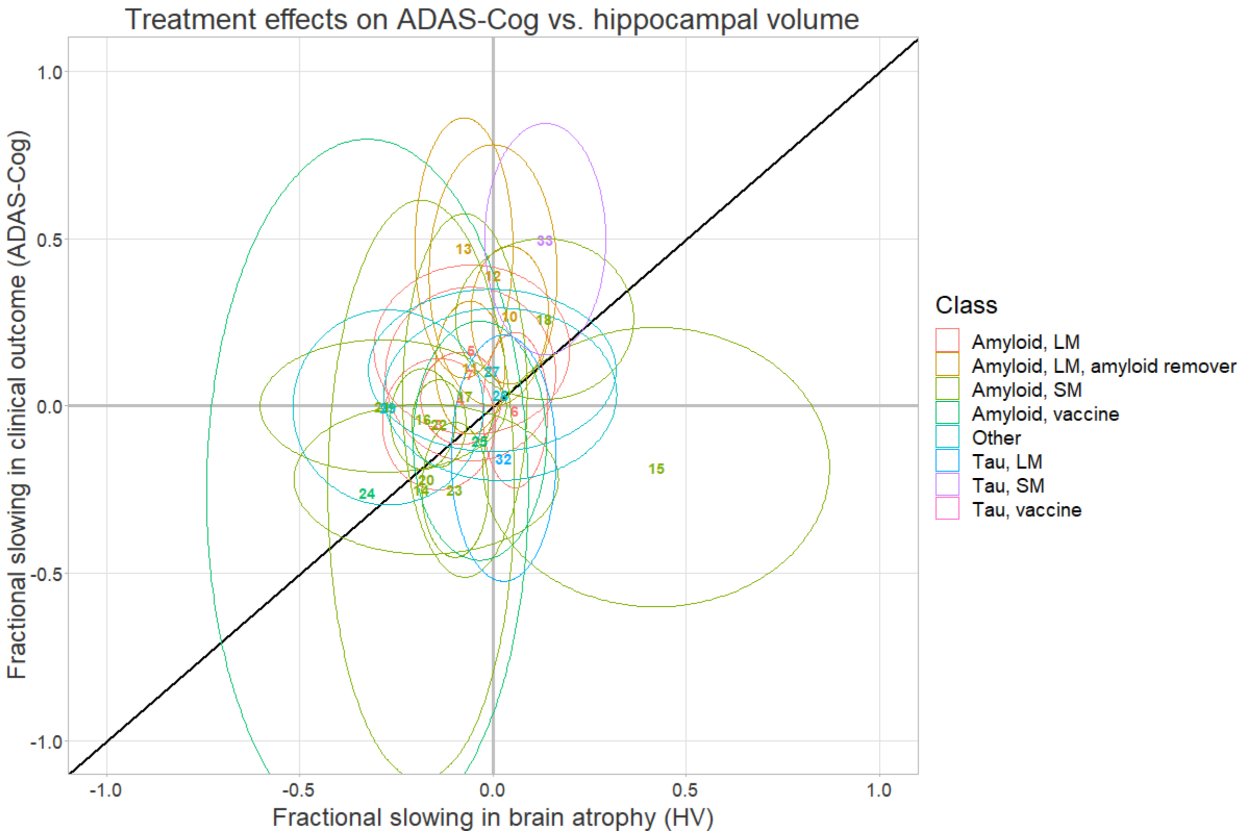

Figure S7: *Scatter plot of treatment effects on data points corresponding to HCV vs. ADAS-Cog.*

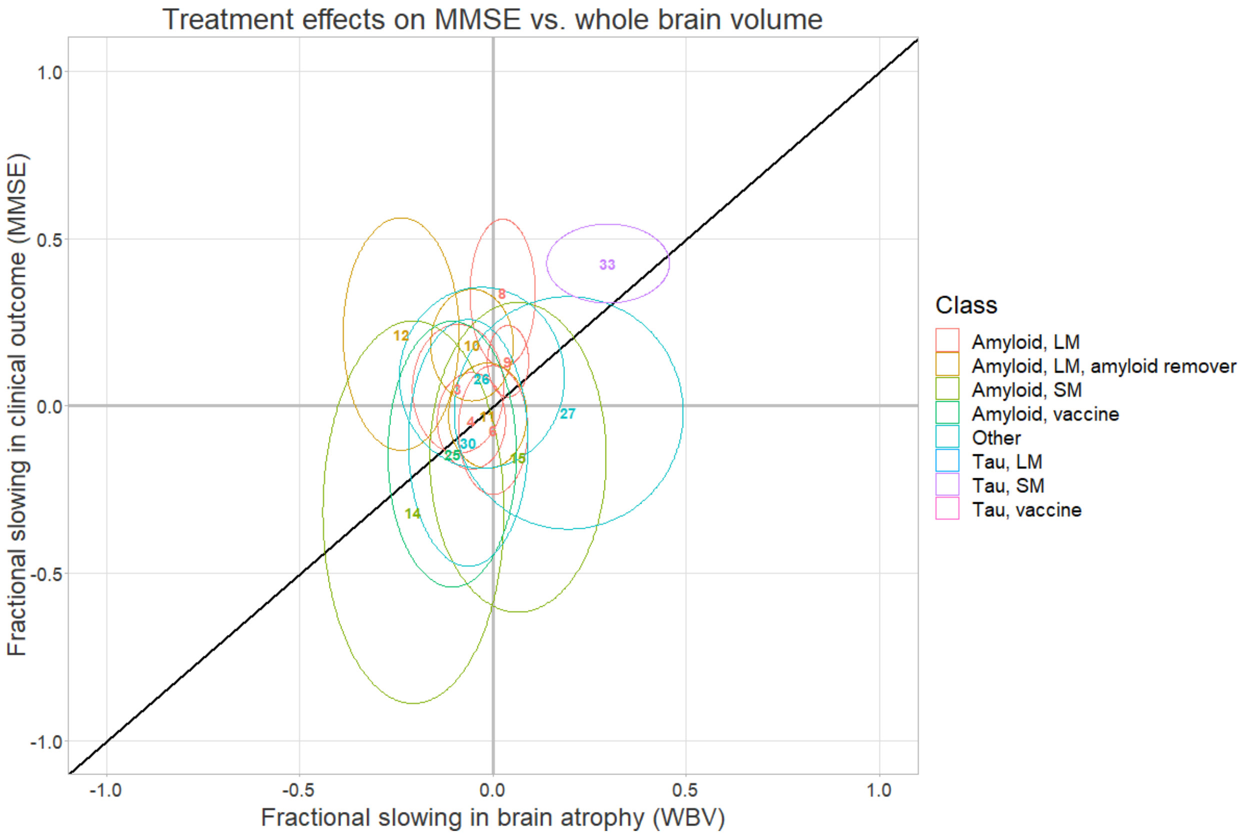

Figure S8: *Scatter plot of treatment effects on data points corresponding to WBV vs. MMSE.*

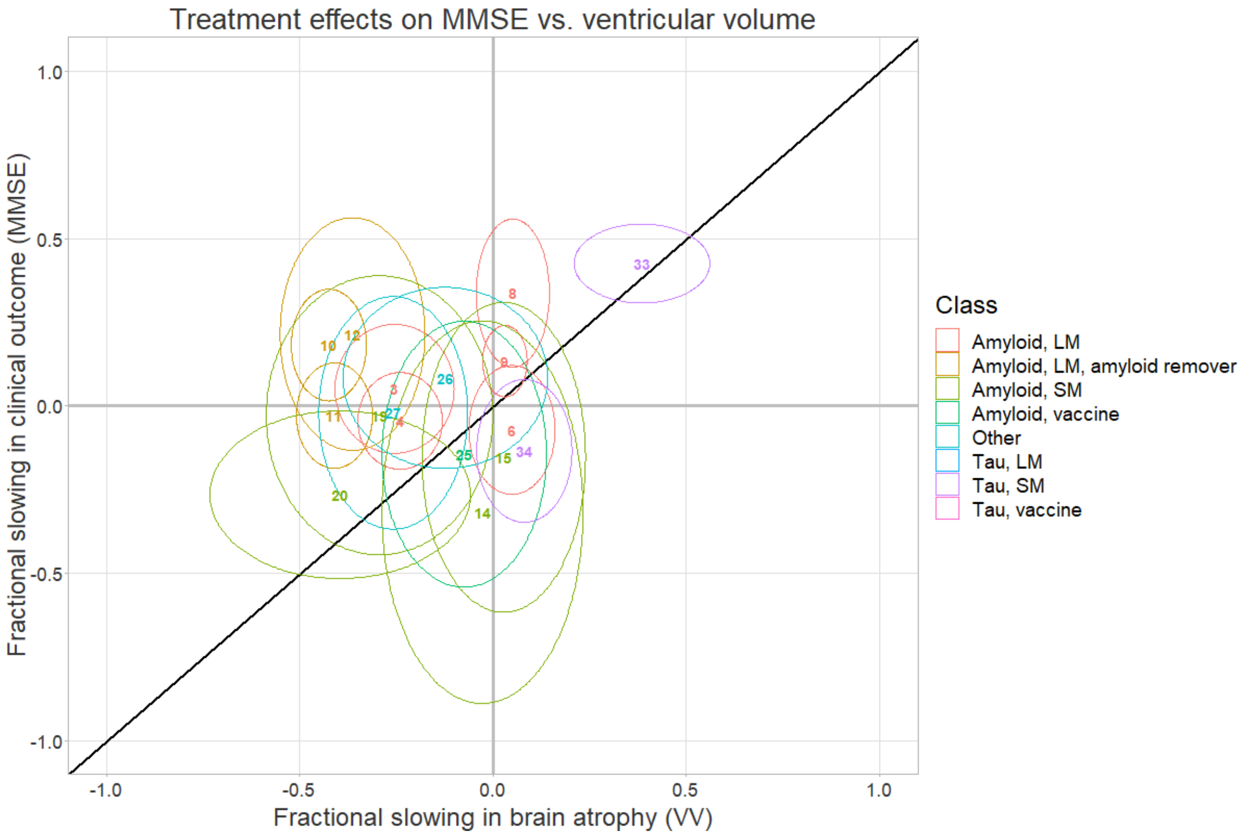

Figure S9: *Scatter plot of treatment effects on data points corresponding to VV vs. MMSE.*

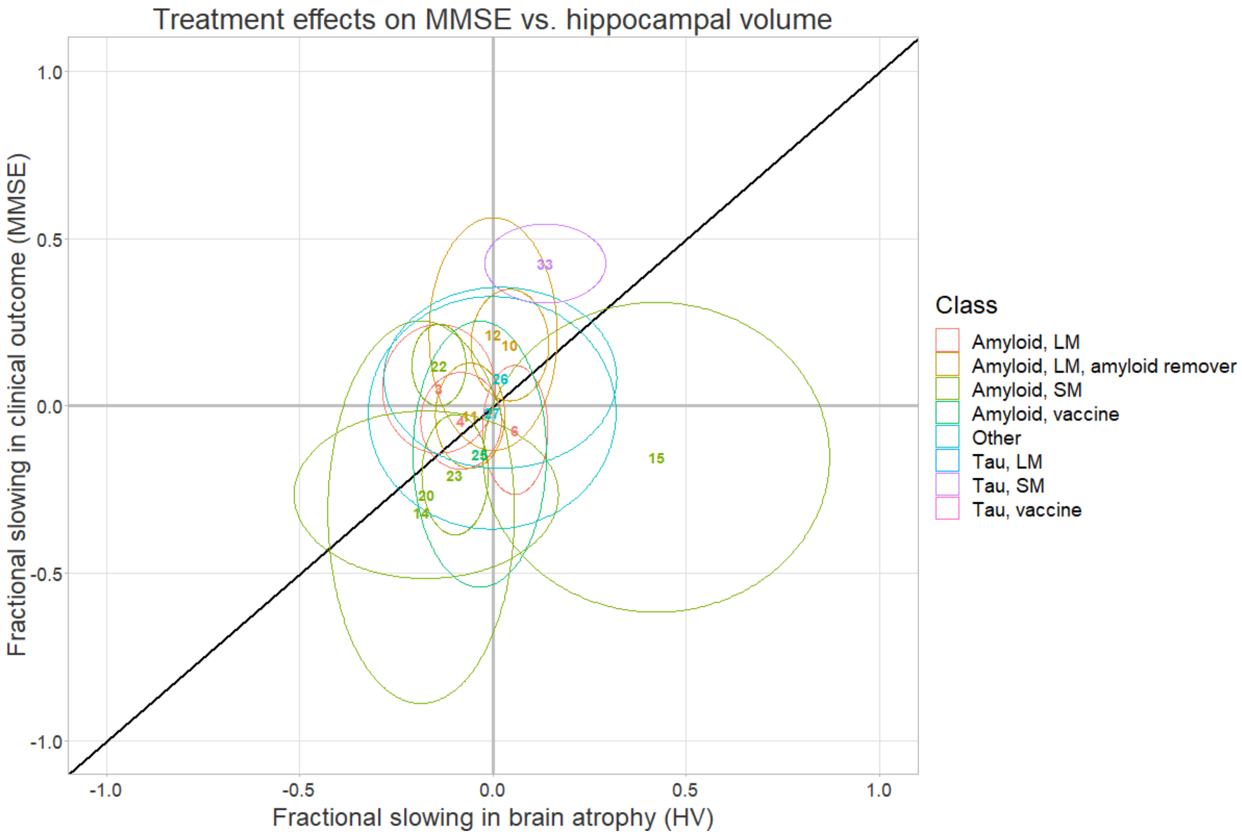

Figure S10: *Scatter plot of treatment effects on data points corresponding to HCV vs. MMSE.*

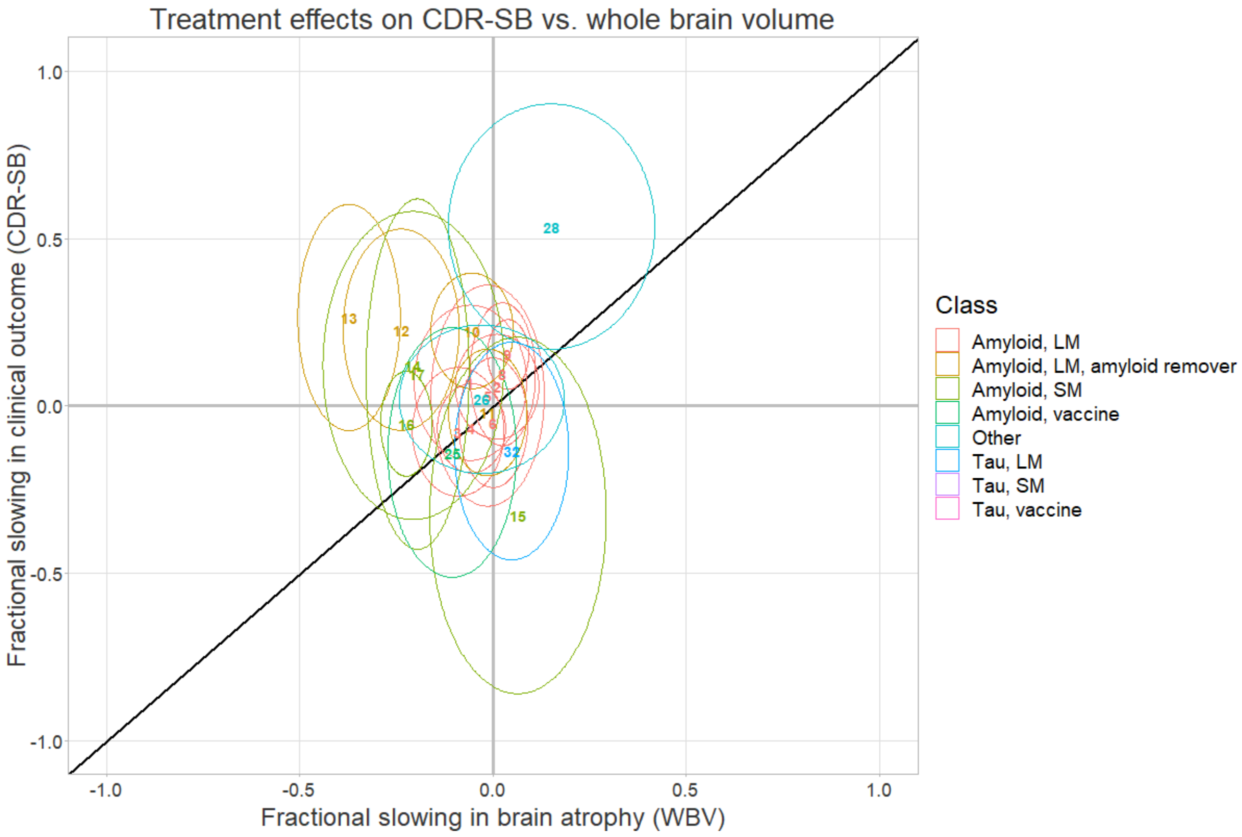

Figure S11: *Scatter plot of treatment effects on data points corresponding to WBV vs. CDR-SB.*

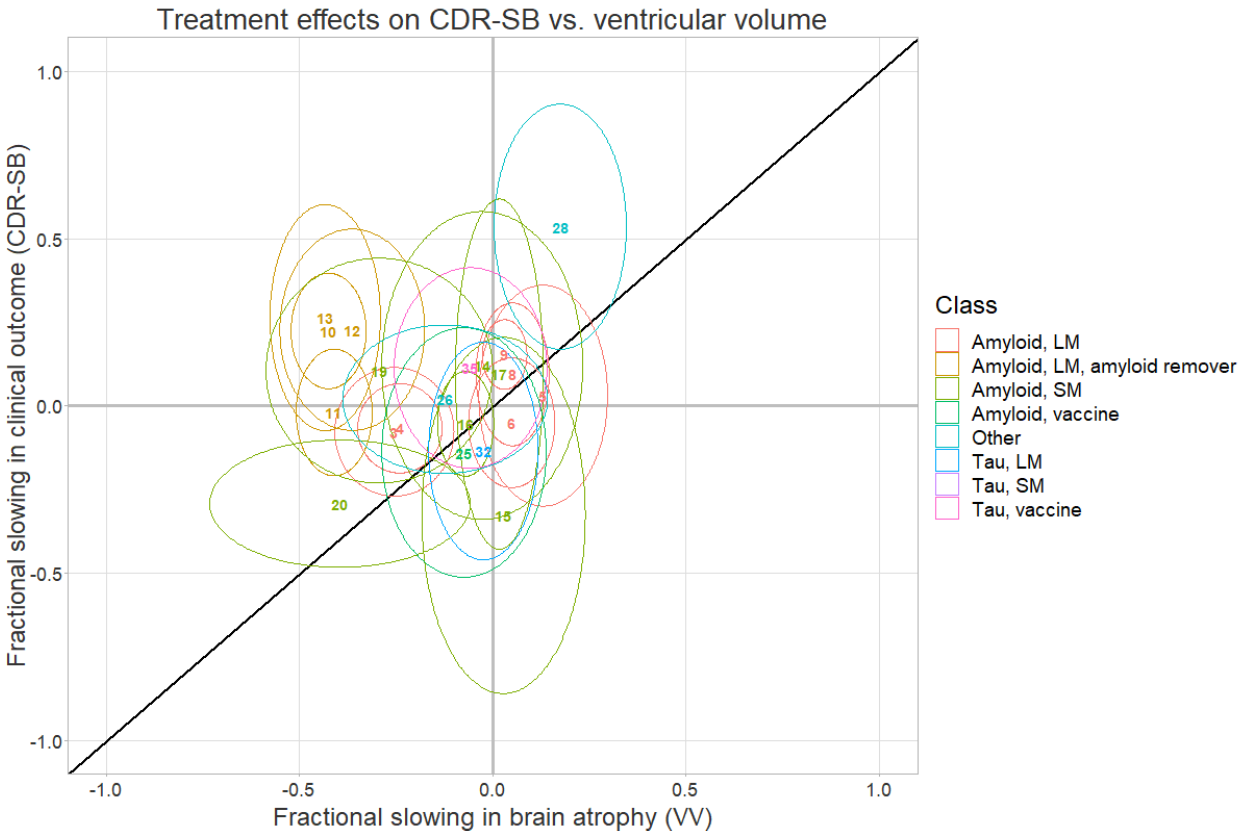

Figure S12: *Scatter plot of treatment effects on data points corresponding to VV vs. CDR-SB.*

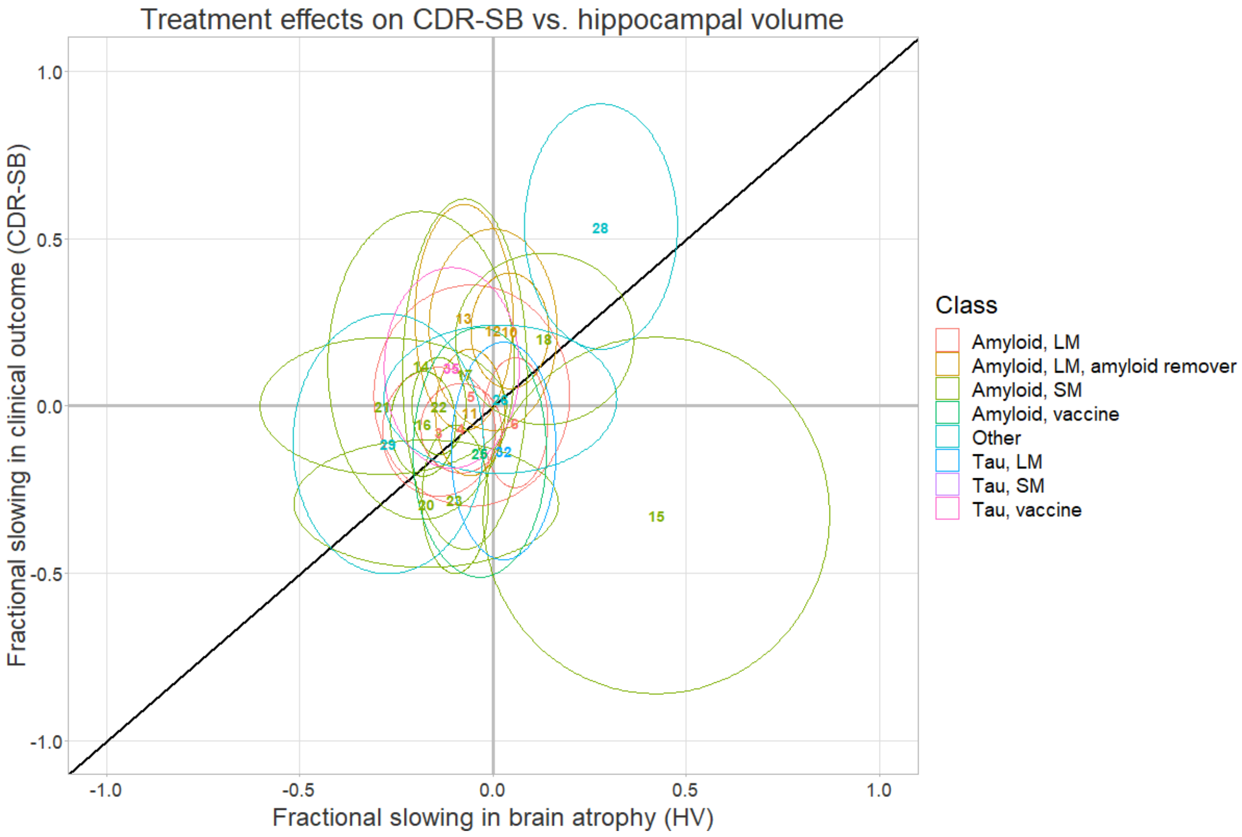

Figure S13: *Scatter plot of treatment effects on data points corresponding to HCV vs. CDR-SB.*
